# Proteomic Analysis of *Fusarium oxysporum*-Induced Mechanism in Grafted Watermelon Seedlings

**DOI:** 10.3389/fpls.2021.632758

**Published:** 2021-03-04

**Authors:** Man Zhang, Jinhua Xu, Runsheng Ren, Guang Liu, Xiefeng Yao, Lina Lou, Jian Xu, Xingping Yang

**Affiliations:** ^1^Jiangsu Key Laboratory for Horticultural Crop Genetic Improvement/Institute of Vegetable Crops, Jiangsu Academy of Agricultural Sciences, Nanjing, China; ^2^School of Life Sciences, Jiangsu University, Zhenjiang, China

**Keywords:** bottle gourd, *Citrullus lanatus*, *Fusarium oxysporum* f.sp. *niveum*, proteomics, rootstock grafting

## Abstract

Grafting can improve the resistance of watermelon to soil-borne diseases. However, the molecular mechanism of defense response is not completely understood. Herein, we used a proteomic approach to investigate the molecular basis involved in grafted watermelon leaf defense against *Fusarium oxysporum* f.sp. *niveum* (*FON*) infection. The bottle gourd rootstock-grafted (RG) watermelon seedlings were highly resistant to *FON* compared with self-grafted (SG) watermelon plants, with a disease incidence of 3.4 and 89%, respectively. Meanwhile, grafting significantly induced the activity of pathogenesis-related proteases under *FON* challenge. Proteins extracted from leaves of RG and SG under *FON* inoculation were analyzed using two-dimensional gel electrophoresis. Thirty-nine differentially accumulated proteins (DAPs) were identified and classified into 10 functional groups. Accordingly, protein biosynthetic and stress- and defense-related proteins play crucial roles in the enhancement of disease resistance of RG watermelon seedlings, compared with that of SG watermelon seedlings. Proteins involved in signal transduction positively regulated the defense process. Carbohydrate and energy metabolism and photosystem contributed to energy production in RG watermelon seedlings under *FON* infection. The disease resistance of RG watermelon seedlings may also be related to the improved scavenging capacity of reactive oxygen species (ROS). The expression profile of 10 randomly selected proteins was measured using quantitative real-time PCR, among which, 7 was consistent with the results of the proteomic analysis. The functional implications of these proteins in regulating grafted watermelon response against *F. oxysporum* are discussed.

## Introduction

Watermelon [*Citrullus lanatus* (Thunb.) Matsum. & Nakai] is an important fruit crop and contributes 11.98% of the world fruit production (FAO, 2018)^1^. *Fusarium* wilt, caused by the soil-borne fungus *Fusarium oxysporum* f. sp. *niveum* (*FON*), is the most serious production-limiting disease in watermelon-growing areas all over the world (Zhang et al., 2015a). The pathogen’s survival in the infected field can be extended for over 10 years (Martyn, 1987) and causes up to 100% yield losses. Resistance to *FON* has been widely described, and resistance locus *Fo-1.3* (Lambel et al., 2014; Meru and McGregor, 2016) to *FON* race 1 and locus *qFon2-2* (Branham et al., 2017) to *FON* race 2 have been identified. Currently, the watermelon reference genome (Guo et al., 2013) and the whole-genome resequencing (Guo et al., 2019) developed a series of potential genes resistant to *Fusarium* wilt. These results should be useful for further elucidating the mechanism of resistance to *Fusarium* wilt and in the development of molecular markers for breeding programs of watermelon.

Grafting is an environmentally friendly and economic technique that is currently being adopted globally in watermelon to cope with soil-borne disease (Lee, 1994). Grafted watermelon was documented with enhanced *Fusarium* wilt resistance (Huh et al., 2002) as well as higher abiotic stress tolerance (Shi et al., 2019) and increased fruit weight and total yield (Davis et al., 2008). Grafting could also facilitate the uptake and utilization of nutrition (Zhang et al., 2012a; Huang et al., 2013). During the interaction between grafted watermelon and *FON*, previous reports have shown changes in physiological (Zhang et al., 2015b) and histological (Zhang et al., 2012b) aspects, indicating that grafting could induce higher levels of defense enzymes and immediately form tyloses in the infected xylem vessels soon after *FON* infection. Ling et al. (2013) found that chlorogenic and caffeic acids in root exudates from rootstock-grafted (RG) inhibited *FON* conidial germination and growth. Further experiment evidenced that grafting can shift the root-secreted protein profile and thus increased *FON* resistance (Song et al., 2016). Recently, a series of comparative proteomic analyses was conducted to explore the cold/chilling (Xu et al., 2016; Shi et al., 2019) or salt (Yang et al., 2012) stress-induced mechanisms in grafted watermelon seedlings. It is noteworthy that most of the previous studies on grafted watermelon resistance assessment were focused on root tissues, whereas few reports have discussed the *FON*-resistant mechanisms mediated by RG plants especially the above-ground leaf tissues. Herein, we hypothesized that the leaf protein profile has been altered after watermelon grafting and plays an essential role in resistance to *Fusarium* wilt.

To gain insight into the molecular mechanisms involved in RG watermelon against *FON* infection, a two-dimensional gel electrophoresis (2-DE) technique in combination with matrix-assisted laser desorption/ionization time-of-flight/time-of-flight mass spectrometry (MALDI-TOF/TOF MS) was used to investigate the above-ground leaf proteome profiles of RG and self-grafted (SG) under *FON* challenge. Proteomic analysis identified 39 differentially accumulated proteins (DAPs), including 11 specific to RG and 4 unique to SG. Thirty-nine DAPs are mainly involved in plant metabolism and energy, defense and stress, protein biosynthesis, and degradation. The expression patterns at the transcriptional level of 10 randomly selected DAPs were validated using real-time PCR. Meanwhile, the functional implications of 39 DAPs especially those specifically accumulated in RG plants were discussed.

## Materials and Methods

### Plant Materials

A *FON*-susceptible watermelon cv. “Sumi 1” (Institute of Vegetable Crops, Jiangsu Academy of Agricultural Sciences, China) and a *FON*-resistant bottle gourd rootstock cv. “Chaofengkangshengwang” (a commercial rootstock from Zhengzhou Fruit Research Institute, Chinese Academy of Agricultural Sciences, China) were used as scion and rootstock, respectively. An “insertion grafting” method (Lee, 1994) was employed to create the grafting combinations. Scions were grafted onto itself (SG) and bottle gourd rootstock (RG), respectively, and the grafting plants were cultivated in a growth chamber at 28/18°C under an 18/6 h light/dark cycle with a relative humidity of 70%. Grafting seedlings at the three-true-leaf stage were used for *FON* inoculation. SG and RG seedlings with *FON* inoculation were named as SG-FON and RG-FON, and their corresponding control seedlings inoculated with distilled water were designated as SG-C and RG-C, respectively. Three replicates were conducted with 30 seedlings for each replication. Ten leaves from various seedlings were pooled for each sample at 240 hours post-inoculation (hpi), when wilting symptoms were observed above the ground and were immediately frozen in liquid nitrogen and stored at −80°C until further use. Three biological replicates were sampled.

### Preparation of Inoculum and Inoculation of Plants

The *FON* race 1 (Institute of Vegetable Crops, Jiangsu Academy of Agricultural Sciences, China) was used for plant inoculation. *FON* was maintained on potato dextrose agar (PDA) at 25°C for 7 days and then inoculated in liquid potato dextrose medium in a 250 ml triangular flask at 25°C on a rotary shaker at 150 rpm for 7 days. Fungal suspension with a concentration of 10^6^ conidia per ml was used to inoculate plants (Chang et al., 2008). For inoculation, grafting seedlings at the three-true-leaf stage were carefully removed from the seedling-raising pot, followed by a rinse with tap water to exclude the soil particles. Roots of the seedlings were immersed in freshly prepared fungal suspension for 30 min, and then seedlings were re-planted in the seedling-raising pot with peat moss + vermiculite + perlite (6/1/3, v/v/v). Seedlings inoculated with sterile distilled water were treated as a control.

### Measurements of Physiological Traits

A total of 0.5 g leaf samples from RG or SG inoculated with *FON* were extracted with 2 ml citrate buffer (0.1 M, pH 5.0) to obtain the crude enzyme extract and used for enzyme activity assay. Chitinase extraction and measurements were performed according to the method described by Boller et al. (1983). In brief, assay mixture (1 ml) containing 0.1 ml of sodium acetate buffer (0.05 M, pH 5.0), 0.3 μM of sodium azide, 1 mg of colloidal chitin, and 0.4 ml of crude enzyme extract was incubated at 37°C for 3 h. Then, 0.4 ml of the assay mixture was mixed with 0.2 ml of sodium borate buffer (0.8 M, pH 9.1) and heated at 100°C for 3 min, followed by added 3 ml of 1% 4-dimethylaminobenzaldehyde (DMAB) solution and incubated for 15 min at 37°C. The absorbance was recorded at 585 nm. Three replicates were conducted.

The β-1,3-glucanase activity was measured as presented by Shu et al. (2006). In brief, assay mixture (1 ml) containing 0.48 ml of sodium acetate buffer (0.1 M, pH 5.2), 1 mg of laminarin, and 0.3 ml of crude enzyme extract was incubated at 50°C for 3 h. Then, 1 ml of 3,5-dinitrosalicylic acid (DNS) was added and heated at 100°C for 5 min. The absorbance was recorded at 540 nm. The amount of glucose was calculated from the standard curve of glucose. Three replicates were conducted.

### Protein Extraction

Total proteins were extracted from leaves of SG and RG seedlings as described by Zhang et al. (2018a). In brief, protein pellets were dissolved in an extraction buffer containing 9.5 M urea, 4% w/v 3-[(3-cholamidopropyl) dimethylammonio]-1-propanesulfonate (CHAPS), 65 mM dithiothreitol (DTT), and 2% v/v immobilized pH gradient (IPG) buffer pH 4–7. The protein concentration was determined using the Bradford method (Bradford, 1976) with bovine serum albumin (BSA) as a standard. Three biological replicates were prepared.

### 2-DE Analysis

Two-dimensional gel electrophoresis was performed using the GE Healthcare 2-DE system as described by Zhang et al. (2018a). Then, 800 μg protein for each sample was loaded to rehydration strip (pH gradient of 4–7, 13 cm) and separated on a Multiphor electrophoresis unit (GE Healthcare, Tokyo, Japan) with the following parameters: 30 V for 12 h, 1 h step from 200 to 500 V, 1 h gradient from 500 to 1,000 V, 30 min gradient from 1,000 to 4,000 V, 30 min gradient from 4,000 to 8,000 V, and 5 h at 8,000 V. After isoelectric focusing, the strips were equilibrated in equilibration buffer I [containing 6 M urea, 50 mM Tris–HCl (1.5 M stocking buffer, pH 8.8), 30% glycerol, 2% sodium dodecyl sulfate (SDS), 1% DTT] with gentle agitation for 15 min, followed by equilibration buffer II [containing 6 M urea, 50 mM Tris–HCl (1.5 M stocking buffer, pH 8.8), 30% glycerol, 2% SDS, 4% iodoacetamide] for 15 min. The strips were transferred to a 12.5% SDS-polyacrylamide gel for protein separation using a Hoefer SE 600 Ruby system (GE Healthcare, Tokyo, Japan). The electrophoresis was carried out at a constant current of 5 mA/gel for 15 min and 10 mA/gel for 6 h.

### Gel Staining and Image Analysis

Gels were stained with Coomassie Brilliant Blue R-250 (Solarbio, Beijing, China). Gel images were acquired using a Typhoon^TM^ 9400 imager (GE Healthcare, Tokyo, Japan) and analyzed by ImageMaster 2D Platinum 6.0 software (GE Healthcare, Tokyo, Japan). For image analysis, manual editing was carried out after automated detection and matching to correct any mismatched and unmatched spots. Proteins were considered to be differentially accumulated if their percent volume ratio was ≥1.5 and ANOVA test value was ≤0.05.

### In-Gel Digestion and MALDI-TOF/TOF MS Analysis

The DAP spots were excised from the 2-DE gel, washed with 50% (v/v) acetonitrile in 0.1 M NH_4_HCO_3_, and digested with modified trypsin (Promega, United States). The digested peptides were further analyzed by MALDI-TOF/TOF MS. MS/MS spectra were searched against the NCBInr database with a Viridiplantae (green plants) restriction and Cucurbitaceae database using the Mascot search tool (Matrix Science, London, United Kingdom).

### Quantitative Real-Time RT-PCR

Total RNA was extracted from leaves of SG and RG seedlings inoculated with *FON* by using an RNApure Plant kit (with DNase I) (CWBiotech, China). Reverse transcription with 1 μg RNA and the oligo dT primer was performed through a BU-Superscript RT kit (Biouniquer, China) according to the manufacturer’s instructions. Real-time PCR was carried out using the 1× SYBR Green PCR Master Mix (PE Applied Biosystems, United States) on the GeneAmp^®^ 7300 Sequence Detection System (PE Applied Biosystems, United States) according to the manufacturer’s instructions. *18SrRNA* (GenBank accession no. AB490410) was used as the internal control for normalization. The relative quantization of gene expressions was calculated using the 2^−ΔΔCT^ method (Livak and Schmittgen, 2001). Three replicates were performed. Primers used in this work were listed in Supplementary Table 1.

### Bioinformatics Analysis

Protein subcellular localization was identified using WoLF PSORT^2^. The possible interaction between the DAPs was carried out through STRING^3^.

## Results

### Comparison of Disease Phenotypes and Physiological Changes of Grafting Seedlings Under *FON* Infection

**FIGURE 1:**
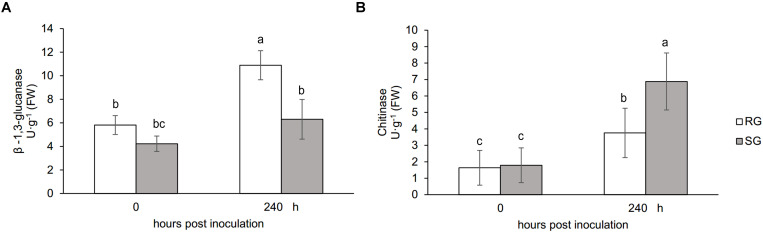
Physiological responses of the grafted watermelon seedlings infected with *FON*. Changes of β-1,3-glucanase **(A)** and chitinase **(B)** activities in the leaves of RG and SG plants. Vertical bars labeled with different letters are significantly different at *p* < 0.05. Error bars are based on three biological replicates.

Plant growth was significantly influenced by bottle gourd rootstock grafting under *FON* infection. SG seedlings exhibited visual wilting symptoms in cotyledons at 240 hpi, whereas RG seedlings grew well, and no cotyledon wilting was observed. RG plants showed high resistance to *FON* with 3.4% of infested plants, whereas SG plants were relatively highly susceptible with 89% of disease incidence at 21 days post-inoculation. *FON* infection induced the accumulation of both β-1,3-glucanase and chitinase, whereas their activities showed differences in RG and SG seedlings. β-1,3-Glucanase was significantly higher in RG than in SG (Figure 1A), whereas chitinase remarkably accumulated more in SG than in RG (Figure 1B). These results indicate that rootstock grafting could improve plants’ resistance by accumulating activity of PR proteases to prevent watermelon seedlings from *FON* infection.

### Proteome Analysis of Grafting Plants With *FON* Infection

**FIGURE 2:**
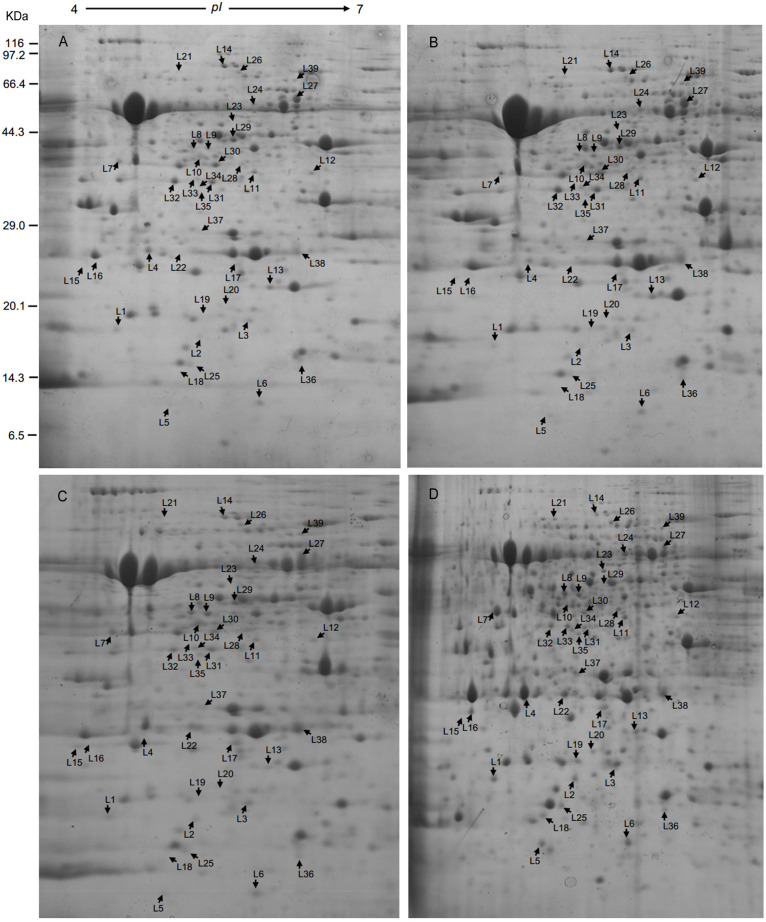
Representative 2-DE gels of proteins extracted from leaves of grafted watermelon seedlings. Proteins extracted from leaves of **(A)** SG-C, **(B)** RG-C, **(C)** SG-FON at 240 hpi, and **(D)** RG-FON at 240 hpi were focused on IPG strips (11 cm, pH 4–7 NL) and separated by SDS-PAGE (12.5%). Arrows mean the differentially accumulated proteins among RG and SG responding to *FON* infection.

**FIGURE 3:**
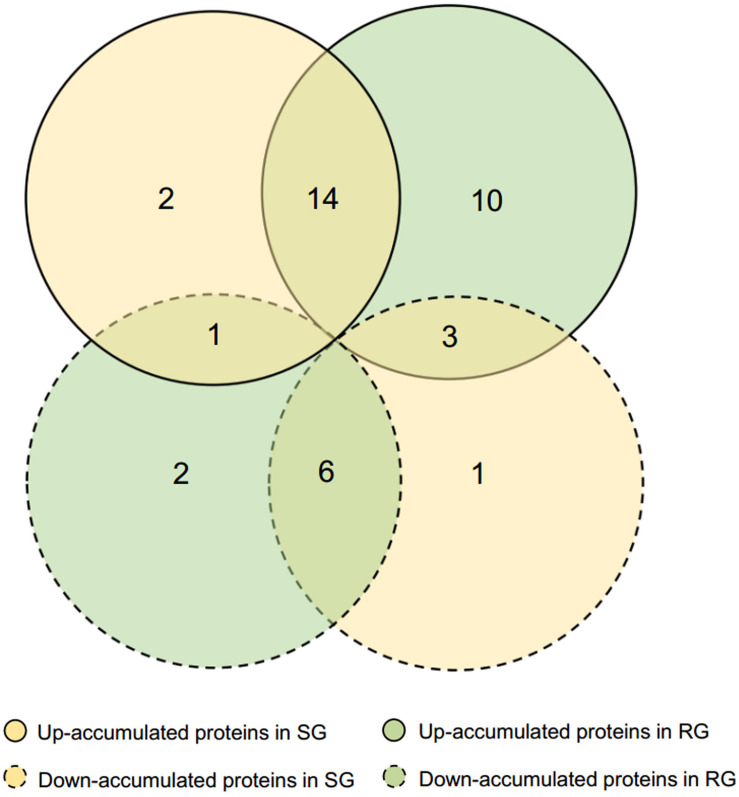
Venn diagram of differentially accumulated proteins (DAPs) in RG and SG infected with *FON*. DAPs were analyzed based on SG-FON vs. SG-C and RG-FON vs. RG-C libraries, respectively.

Leaf samples of RG and SG seedlings inoculated with *FON* at 240 hpi were analyzed to investigate the *FON* responsive proteins using 2-DE. More than 1,340 protein spots were generated on each gel, and 917 were reproducibly detected. Proteins with fold change >1.5 at *p*-value < 0.05 were considered to be differentially accumulated (DAP). Accordingly, 39 DAPs were identified (Table 1 and Figures 2, 3), of which 14 up-accumulated proteins were shared in both RG and SG; 10 and 2 proteins were up-accumulated only in RG and SG, respectively. Six down-accumulated proteins were overlapped in both RG and SG; 1 and 2 proteins were down-accumulated only in RG and SG, respectively (Figure 3). Three proteins were up-accumulated in RG but down-accumulated in SG; 1 protein was up-accumulated in SG but down-accumulated in RG (Figure 3).

**TABLE 1:** Identification of differentially accumulated proteins in leaves of grafted watermelon under *FON* infection.

^a^Spot ID	Watermelon accession no.	Protein description	^b^Mr (KD)/pI	^c^SC (%)	Score	Cell compartment	^d^Abundance vol%
**C metabolism**							
L14	Cla97C05G082860	Transketolase, putative	80.62/6.37	15	272	Cytoplasm	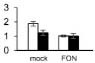
L26	Cla97C09G167630	Beta-xylosidase 2	83.30/8.63	8	227	Vacuole	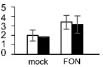
L33	Cla97C02G032410	Malate dehydrogenase	43.60/7.75	33	380	Chloroplast	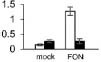
**N metabolism**							
L8	Cla97C09G166940	Glutamine synthetase	39.19/5.99	14	365	Cytoplasm	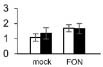
L9	Cla97C05G093490	Cytosolic glutamine synthetase	39.22/5.90	11	313	Cytoplasm	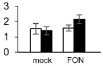
**ROS metabolism**							
L7	Cla97C02G045130	Peroxidase	34.58/8.32	36	283	Chloroplast	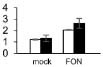
L17	Cla97C09G162960	Germin-like protein	21.51/6.50	29	291	Chloroplast	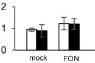
L23	Cla97C08G148570	L-Ascorbate peroxidase T	49.69/7.96	9	274	Chloroplast	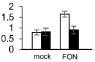
L37	Cla97C02G046770	L-Ascorbate peroxidase	27.78/5.82	9	245	Chloroplast	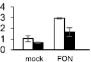
**Energy metabolism**							
L13	Cla97C08G146490	ATP synthase subunit delta, chloroplastic	25.77/9.78	11	218	Mitochondria	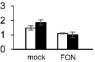
L24	Cla97C04G076580	Enolase	47.77/5.55	13	343	Cytoplasm	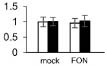
L27	Cla97C02G030740	ATP synthase subunit alpha	54.26/4.69	36	569	Cytoplasm: cytoskeleton	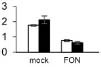
L30	Cla97C03G055870	ATP synthase gamma-subunit	41.25/5.99	21	492	Chloroplast	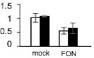
**Protein biosynthetic**							
L1	Cla97C05G084830	Cyanate hydratase	18.89/6.53	25	100	Cytoplasm	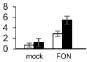
L10	Cla97C11G219440	Cysteine synthase	40.97/7.46	22	165	Chloroplast	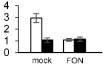
L11	Cla97C02G049790	Enoyl reductase	40.91/9.57	13	414	Chloroplast	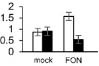
L12	Cla97C07G132520	60S acidic ribosomal protein P0	34.21/4.77	17	165	Cytoplasm	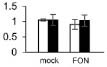
L21	Cla97C10G187570	Hsp70–Hsp90 organizing protein 3-like	65.14/5.91	8	249	Cytoplasm	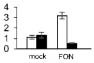
L25	Cla97C08G153070	6,7-Dimethyl-8-ribityllumazine synthase	24.20/8.09	19	157	Chloroplast	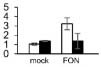
L28	Cla97C06G109750	Arginase	47.99/7.36	20	380	Chloroplast	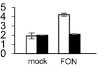
L29	Cla97C06G127160	Elongation factor Tu, chloroplastic	51.24/6.37	25	779	Chloroplast	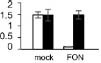
L34	Cla97C05G092190	Sulfurtransferase	49.09/9.22	15	151	Chloroplast	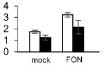
L39	Cla97C02G042600	RNA-binding KH domain-containing protein	67.74/6.00	18	341	Cytoplasm	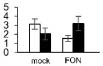
**Defends and stress**							
L2	Cla97C05G088120	Universal stress protein	18.08/5.96	34	135	Cytoplasm	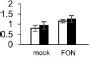
L4	Cla97C11G207000	Jasmonate-induced protein	23.69/6.39	37	314	Cytoplasm	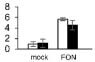
L6	Cla97C08G158200	Thioredoxin h	12.99/5.62	57	235	Cytoplasm	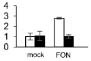
L15	Cla97C01G003090	Thaumatin-like protein	24.49/6.97	26	194	Chloroplast	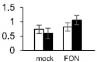
L16	Cla97C01G003090	Thaumatin-like protein	24.49/6.97	36	237	Chloroplast	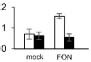
L22	Cla97C11G207000	Jasmonate-induced protein	23.69/6.39	26	315	Cytoplasm	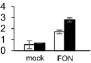
L35	Cla97C02G034000	Isoflavone reductase-like protein/	33.75/7.77	32	321	Cytoplasm	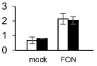
**Translation**							
L3	Cla97C05G097300	Eukaryotic translation initiation factor 5A (eIF5A)	17.56/5.87	35	220	Cytoplasm	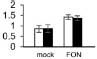
L20	Cla97C05G084840	Multiple organellar RNA editing factor 2	27.26/8.97	17	102	Mitochondria	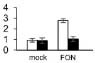
**Photosystem**							
L31	Cla97C10G185280	Ferredoxin–NADP reductase	40.30/8.17	23	371	Chloroplast	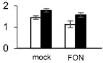
L32	Cla97C10G185280	Ferredoxin–NADP reductase	40.30/8.17	20	336	Chloroplast	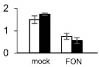
L36	Cla97C11G213660	Thylakoid lumenal 15 kDa protein 1, chloroplastic	22.61/6.52	27	373	Chloroplast	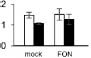
L38	Cla97C07G137540	Oxygen-evolving enhancer protein 2, chloroplastic	28.35/8.04	24	256	Chloroplast	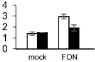
**Signal transduction**							
L18	Cla97C05G080010	Nucleoside diphosphate kinase	16.43/6.81	42	418	Cytoplasm	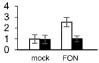
**Transport**							
L5	Cla97C07G142420	Nuclear transport factor 2	13.51/6.50	34	168	Nucleus	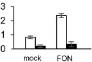
L19	Cla97C02G027850	Cytochrome b6-f complex iron–sulfur subunit	24.22/8.28	23	421	Chloroplast	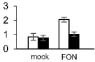

*^a^ Spot ID corresponding to spots in Figure 2. ^b^Mr, molecular weight; pI, isoelectric point. ^c^SC, sequence coverage. ^d^The relative volumes of three replicates (±SD) are presented. White column means RG seedlings; black column means SG seedlings. From left to right, samples were RG-C, SG-C, RG-FON, and SG-FON.*

### Classification of *FON* Responsive DAPs

**FIGURE 4:**
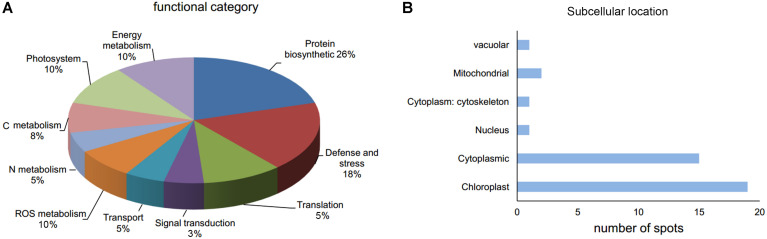
Functional category **(A)** and subcellular location **(B)** of DAPs in leaves of grafted watermelon in response to *FON* infection.

Thirty-nine DAP spots were excised from the gel and subjected for MALDI-TOF/TOF MS analysis. All the 39 DAPs were successfully identified (Table 1), of which 90% of proteins were identified with >10% sequence coverage showing high confidence. The DAPs were functionally categorized mainly in 10 different pathways: C metabolism-related (3), N metabolism (2), energy metabolism (4), protein biosynthetic (10), photosystem (4), defense and stress (7), reactive oxygen species (ROS) metabolism (4), translation (2), signal transduction (1), and transport (2) (Table 1 and Figure 4A). In addition, it was reported that subcellular localization of the proteins was closely related to their physiological functions in plants (Sun et al., 2014). Thirty-nine DAPs were further localized to the chloroplast (19), cytoplasm (15), mitochondria (2), nucleus (1), cytoskeleton (1), and vacuole (1) (Figure 4B), indicating that chloroplast located proteins (49%) play crucial roles in plant defense against *FON* in grafting seedlings.

### Protein–Protein Interaction Network

**FIGURE 5:**
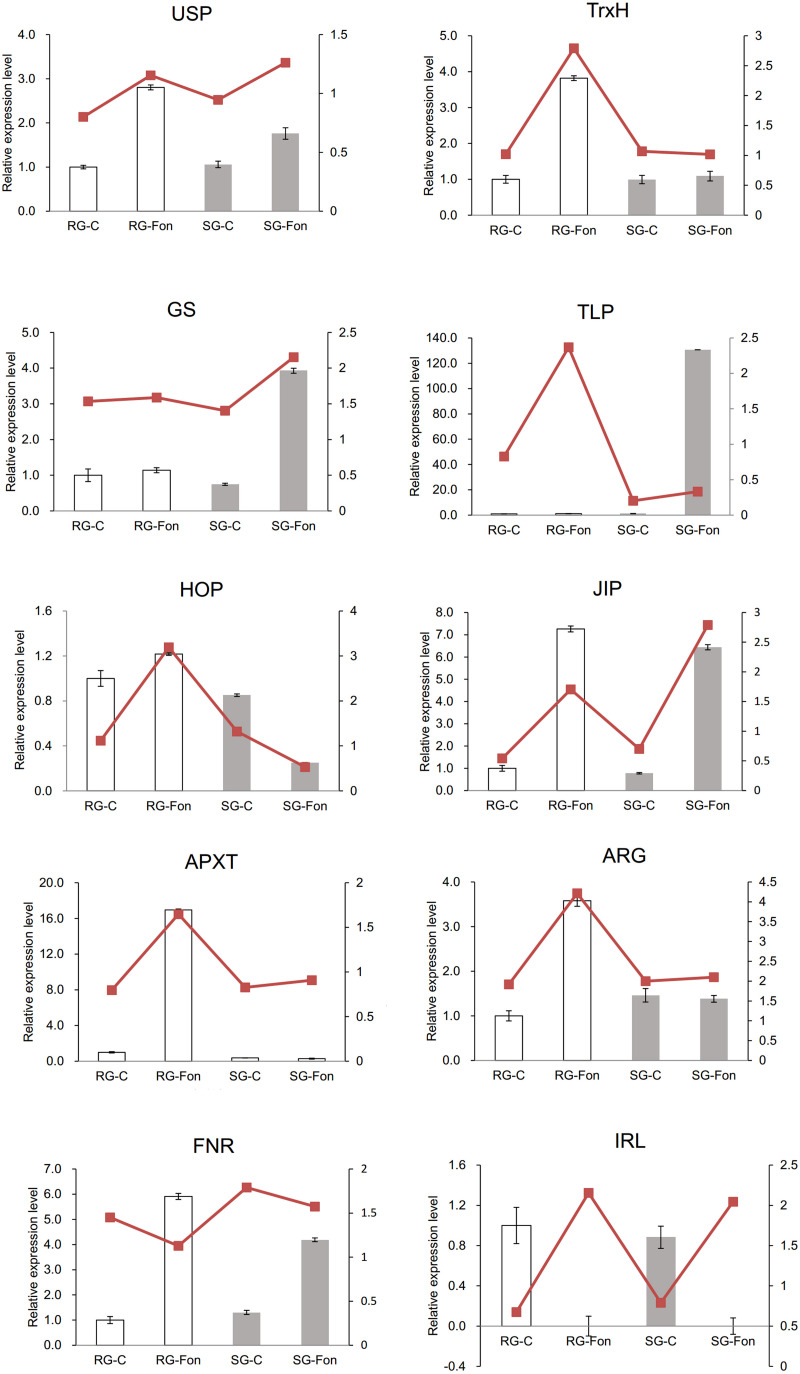
Protein–protein interaction (PPI) network as elucidated through the STRING 11.0 online software with a confidence score of 0.4 using *Arabidopsis thaliana*. The network nodes represent proteins, and the edges indicate the predicted functional associations. The clusters mean the highly interacting proteins involved in photosystem, energy metabolism, translation, and C metabolism.

A predicted protein–protein interaction network was generated using STRING 9.0 to reveal functional links between proteins differentially accumulated in watermelon leaves in response to *F. oxysporum* infection. As expected, one protein interacted with another one to constitute a complex interaction network (Figure 5 and Supplementary Table 2). The main cluster revealed strong interaction among TAPX-FNR1-RABE1b-LOS2-MDH. These proteins belong to ROS metabolism, photosystem, translation, energy metabolism, and C metabolism. This observation indicated that these proteins function cooperatively to prevent plants from *FON* infection in grafted watermelon seedlings.

### Analysis of the Expression Profiles of the mRNAs of Some Identified Proteins by Real-Time PCR

**FIGURE 6:**
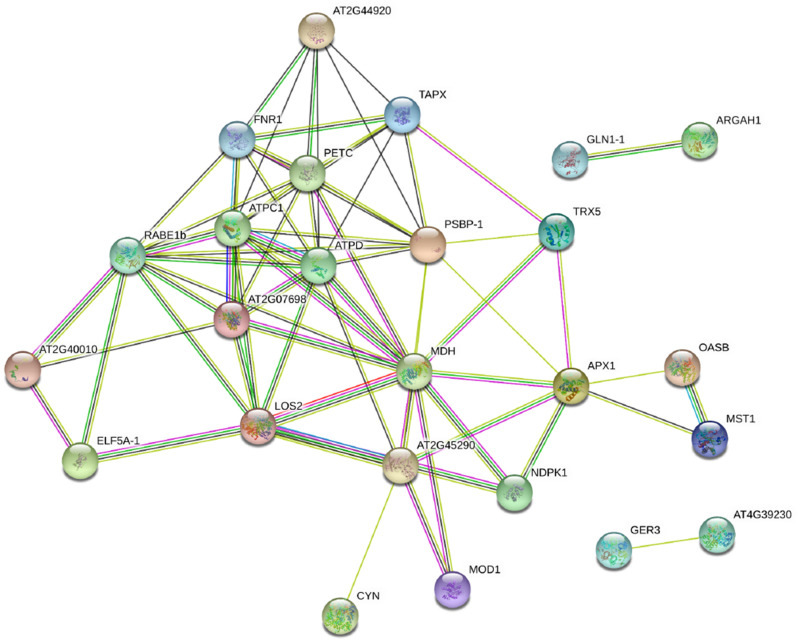
Relative expression level of genes corresponding to 10 randomly selected DAPs in leaves of grafted watermelon in response to *FON* infection. The value of the relative expression level was normalized to *18SrRNA*. Error bars were based on three technical replicates. USP (spot L2), universal stress protein; TrxH (spot L6), thioredoxin h; GS (spot L9), glutamine synthetase; TLP (spot L15), thaumatin-like protein; HOP (spot L21), Hsp70–Hsp90 organizing protein; JIP (spot L22), jasmonate-induced protein; APXT (spot L23), L-ascorbate peroxidase T; ARG (spot L28), arginase; FNR (spot L31), ferredoxin–NADP reductase; IRL (spot L35), isoflavone reductase-like protein.

Ten DAPs were selected for transcript level to validate the proteomic data. In our proteome work, protein spots L2 (USP), L22 (JIP), and L35 (IRL) were up-accumulated in both SG and RG. Protein spot L31 (FNR) was down-accumulated in both SG and RG. Protein spot L21 (HOP) was up-accumulated in RG but down-accumulated in SG. Protein spots L6 (TrxH), L23 (APXT), and L28 (ARG) were only up-accumulated in RG but no response in SG. Protein spots L9 (GS) and L15 (thaumatin-like protein, TLP) were only up-accumulated in SG but no response in RG. Quantitative reverse transcription-PCR (qRT-PCR) (Figure 6) showed that the gene transcription level of seven proteins (USP, TrxH, GS, HOP, JIP, APXT, and ARG) was correlated with MS-based level supporting the reliability of the proteomic approach, whereas the gene expression profile of the other three proteins (TLP, FNR, and IRL) was not correlated with their protein level.

## Discussion

### Energy and Metabolism Proteins

Defense mechanisms are employed to minimize pathogenic damage, among which plant metabolism and energy supply are the key factors for the defense of plants against pathogens (Zaynab et al., 2019). Pathogen infection leads to dramatic changes in the carbohydrate metabolism of the infected plant tissue that was supported in our work by the alteration of C metabolism-related proteins (spots L14, L26, L33) in grafted watermelon seedlings infected with *FON*. Specifically, the abundance of malate dehydrogenase (MDH, spot L33) was up-accumulated extremely in RG under *FON* challenge. MDH is the key tricarboxylic acid (TCA) cycle enzyme that reversibly catalyzes the interconversion of malate and oxaloacetate (Musrati et al., 1998). Many studies have evaluated the function of MDH in plant defense against various abiotic stresses either by maintaining energy homeostasis (Selinski et al., 2014) or by enhancing organic acid synthesis (Tesfaye et al., 2001). We therefore speculated that the improved *FON* tolerance of RG may be related to the up-representation of MDH (spot L33) that enhanced the TCA cycle.

Photosynthesis is one of the plant metabolisms that could be involved in defense against pathogens. Most studies have shown that pathogen invasion locally reduced the rates of photosynthesis that could be interpreted as for freeing up resources utilized for the defense response (Somssich and Hahlbrock, 1998) or protecting the photosynthetic apparatus against light-induced damage (Niyogi, 2000). In contrast, stimulated rates of photosynthesis have been described in several compatible plant–pathogen interactions. As an example, in the tomato plants infected with *Pseudomonas syringae* and *Botrytis cinerea*, Berger et al. (2004) found the expected inhibition of photosynthesis in the infection sites as well as distinct stimulation of photosynthesis in the surrounding circular areas. The authors presumed that the enhanced photosynthesis could be part of the defense strategy for plants to produce assimilates for defense reactions that ultimately helps to confine pathogen growth. In this study, three ATP synthase proteins (spots L13, L27, L30) and two ferredoxin–NADP reductase (FNR) proteins (spots L31, L32) were downregulated in abundance in both RG and SG plants in response to *FON* infection. One of the possible explanations might be the plant’s attempt to maintain the intactness of the membranes or repair them for carrying on the electron transport reactions (Dinakar et al., 2012).

### Proteins Involved in Defense and Stress

Activation of plant defense systems was evidenced by an altered abundance of defense-related proteins, such as universal stress protein 1 (spot L2), jasmonate-induced protein (spots L4, L22), isoflavone reductase-like protein (spot L35), and pathogenesis-related protein PR-5 (TLP, spots L15, L16). The density of TLP (spot L15) was up-accumulated in SG, whereas there remained no changes in RG. Conversely, the abundance of TLP (spot L16) was significantly activated in RG, whereas it was repressed in SG. A wealth of evidence suggests that the accumulation of TLPs induces systemic acquired resistance that boosts the plant’s resistance against fungal pathogens (Dong, 2001). Sun et al. (2020) proved that TLP performs different functions in other physiological processes. They found that Pe-TLP played a role as an elicitor of other anti-fungal proteins that cause the activation of other defense pathways. Meanwhile, fungal pathogens employed different mechanisms simultaneously or in succession to facilitate successful infection during the plant–fungus interaction. It was proven that TLP could either be bound to *Trichoderma virens* Alt a 1 protein (Kumar and Mukherjee, 2020) or be absorbed by fungal mycelia (Marcato et al., 2017) and hence suppressing plant defense. Based on these observations, we presume that different mechanisms may be deployed during the interaction of grafted watermelon and *FON*.

Among ROS-scavenging enzymes, thioredoxin h (spot L6) and peroxidase (spot L7) were identified in grafted watermelon leaves infected with *FON*. The abundance of these proteins was up-accumulated while exhibiting different profiles in RG and SG seedlings. Thioredoxin h (spot L6) was observed to be up-represented in RG seedlings, whereas there remained no changes in SG. A series of reports documented that increased production of thioredoxin h was triggered by accumulation of ROS and misfolded proteins in ROS homeostasis in defense against fungal (Zhang et al., 2017) and viral infections (Das et al., 2019) and abiotic stress (Shi et al., 2019). Thioredoxin h functions in the protection of infected plants against oxidative stress by reducing disulfide bonds on selected target proteins (Jacquot et al., 1997) or by acting as a molecular chaperone for peroxisome matrix proteins as well as antioxidant in peroxisome (Du et al., 2015). Based on these observations, elevated thioredoxin h protein in RG might be the result of maintaining ROS homeostasis in defense of RG watermelon plants against *FON* and thus allows the RG seedlings to survive.

The abundance of peroxidase (spot L7) was higher in SG than in RG (Table 1 and Figure 1), indicating that SG seedlings need more peroxidase to reduce the ROS accumulation in their intracellular system (Das et al., 2019). Peroxidase activity was also proven as key players particularly in cell wall modifications by catalyzing lignification (Passardi et al., 2004), implicating that structures were produced around the sites of potential *FON* ingress to establish physical barriers to prevent the progress of pathogen in SG plants (Zaynab et al., 2019). Additionally, the differential representation of antioxidant proteins was observed in grafted watermelon infected with *F. oxysporum*. Two L-ascorbate peroxidase (APX, spots L23, L37) were identified. APX removes potential harmful H_2_O_2_ from plant cells by detoxificating H_2_O_2_ into water utilizing ascorbate as electron donor (Shigeoka et al., 2002). Overproduction of APX in this work might be the result of the enhancement of active oxygen scavenging system in grafted watermelon (Sarowar et al., 2005) and thus result in *F. oxysporum* resistance. However, other studies have suggested that chloroplastic APX is highly sensitive to inactive to excessive ROS, namely, high contents of ROS under extreme stress conditions may repress APX, which was interpreted as that APX deficiency could activate a compensatory mechanism to protect plants against oxidative stress (Caverzan et al., 2014) and therefore explained the low density of APX (spot L23) in SG plants in this work.

Germin-like protein (GLP) is a ubiquitous water-soluble glycoprotein characterized by various enzymatic activities and is known to play crucial roles in plants’ resistance to fungal pathogens due to its antioxidant potential (Wang et al., 2013). *GLP*-overexpressing plants exhibited enhanced resistance to bacterial blight and fungal pathogens, which was explained by promoting ROS accumulation (Beracochea et al., 2015) or regulating the expression of plant defense-related genes (Liu et al., 2016) in transgenic plants. The up-representation of GLP (spot L17) in the current work suggests that GLP functions as a positive regulator of grafted watermelon resistance to *FON*.

### Protein Biosynthesis and Degradation

Changes in protein synthesis and degradation were evidenced by the altered abundance of 10 proteins (Table 1). Earlier reports showed the increased amino acid triggered plants’ resistance to salt stress resulting from the strong nitrogen uptake ability by the root system of the bottle gourd rootstock (Martinez-Ballesta et al., 2010). In our study, two proteins, cyanate hydratase (spot L1) and sulfurtransferase (spot L34), were up-accumulated in both RG and SG; four proteins (spots L11, L21, L25, L28) were up-represented specifically in RG, whereas they were down-accumulated in SG, especially for Hsp70–Hsp90 organizing protein (HOP/Sti 1 spot L21). HOP/Sti 1 is a co-chaperone that could bind both Hsp70 and Hsp90 chaperones and enables complex formation at the same time. HOP/Sti 1 functions in host physiological processes linked to disease states and roles in aiding protein folding and avoiding or rescuing misfolded proteins (Tiroli-Cepeda and Ramos, 2011). The differential alteration of HOP/Sti 1 protein abundance (spot L21) in this work implicates its crucial role in maintaining proteostasis in RG and SG under *F. oxysporum* challenge.

Four proteins (spots L10, L39, L12, L29) involved in protein synthesis were under-represented in both RG and SG. EF-Tu (spot 29) was suppressed strikingly in RG, whereas it remained unchanged in SG. EF-Tu plays a vital role in mRNA decoding by proofreading each aminoacyl-tRNA (Morse et al., 2020). Although speculation exists that EF-Tu is involved in the other cell functions (Choi et al., 2000), the contribution of the lower abundance of EF-Tu to the RG plant’s resistance to *F. oxysporum* remains to be explored.

### DAPs Involved in Signal Transduction

The abundance of a nucleoside diphosphate kinase (NDPK, spot L18) was identified to be significantly up-represented in RG infected with *F. oxysporum*. A similar result was observed in tolerant *Arabidopsis thaliana* line upon challenge with *Alternaria brassicae* (Sharma et al., 2007). The authors speculated the *NDPK* role in mediating plant defense to *A. brassicae via* the ROS-mediated signaling pathway. NDPK, a ubiquitous and highly conserved enzyme, plays a role in the primary metabolism for maintaining the nucleotide balance in the cell (Sweetlove et al., 2001), as well as functions in signal transduction pathway for mediating plant defense against abiotic stress (Moon et al., 2003), hormone responses (Galvis et al., 2001), and pathogen (Sharma et al., 2007). Overexpression of *AtNDPK2* reduced the accumulation of ROS and thereby conferred enhanced tolerance to multiple environmental stresses (Moon et al., 2003). Further evidence showed that the enhancement of plant defense resulted from the association of *AtNDPK2* with H_2_O_2_-mediated MAPK signaling in plants. Our current observation that NDPK (spot L18) abundance is strikingly increased in RG suggests that NDPK appears to play a vital regulatory role *via* ROS-mediated signaling in mediating RG plants’ response to *FON* challenge.

### Translation and Transport-Related Proteins

Two proteins, eukaryotic translation initiation factor 5A (eIF5A, spot L3) and multiple organellar RNA editing factor 2 (MORF2, spot 20), associated with translation were identified in grafted watermelon infected with *FON*. The abundance of eIF5A (spot L3) was up-accumulated in both RG and SG plants under pathogen challenge. eIF5A is involved in the initial process of protein translation and can be induced by the pathogen (Campo et al., 2004). In the present work, the up-representation of eIF5A (spot L3) following *F. oxysporum* challenge may be responsible for regulating proteins important for pathogen attack. Rather, MORF2 up-represented extremely in RG while with low intensity in SG. MORF2 is an essential component of the plant RNA editosome and is a major player as an editing factor regulating the RNA editing efficiency at multiple sites (Bentolila et al., 2013). The function of MORF2 during plant and pathogen interaction remains unknown, although observation exists that plastid-signaling defective mutant *gun1*, directly interacting with MORF2, showed a deficiency of rapid RNA multiplication that results in more severe oxidative stress (Zhang et al., 2011).

A nuclear transport factor 2 (NTF2, spot L5) protein was identified in this work as shown to be markedly accumulated in RG, whereas it remained unchanged in SG under *FON* challenge. The up-representation of NTF2 was observed previously in the compatible interaction of *Verticillium dahliae* with tomato (Hu et al., 2019). Furthermore, the silencing of *TaNTF2* reduced the resistance of wheat to different avirulent isolates of the *Puccinia striiformis*, which speculated that NTF2 acted as a critical regulator in the Ran-mediated signal transduction in the plant immune system (Zhang et al., 2018b).

## Conclusion

In conclusion, we reported here that bottle gourd rootstock grafting can significantly improve watermelon resistance against *FON.* Proteomic analysis revealed 39 DAPs in leaves of grafted watermelon plants under *FON* challenge. These reprogrammed proteins were classified into 10 different biological functional categories, among which, protein biosynthesis (26%) and defense and stress (18%) are the two major affected biological functional processes, indicating that they act as a positive regulator in defense responses triggered by *FON* in grafted watermelon. Notably, we identified six proteins, i.e., MDH (spot L33), HOP/Sti 1 (spot L21), MORF2 (spot L20), NTF2 (spot L5), NDPK (spot L18), and thioredoxin h (spot L6) were accumulated in abundance only in RG in response to *FON* infection. These proteins presented in different functional categories while interacted with each other closely and play crucial roles in rootstock grafting-mediated resistance. These specifically accumulated proteins will be further characterized to elucidate their roles in rootstock grafting-induced resistance during watermelon and *FON* interaction. Overall, the proteomic data provide us with new insight into a better understanding of the molecular defense mechanisms in rootstock-grafted watermelon.

## Data Availability Statement

The datasets presented in this study can be found in online repositories. The names of the repository/repositories and accession number(s) can be found in the article/Supplementary Material.

## Author Contributions

XYN and MZ designed the experiments. MZ performed 2-DE and SDS-PAGE experiment. RR, LL, and JiaX contributed to the data analysis. GL and XYO contributed to the enzyme activity analysis. JinX and XYN contributed to revise the manuscript. All authors contributed to the article and approved the submitted version.

## Conflict of Interest

The authors declare that the research was conducted in the absence of any commercial or financial relationships that could be construed as a potential conflict of interest.

## Footnotes

1 http://www.fao.org/faostat/en/

2 https://wolfpsort.hgc.jp/

3 https://string-db.org/

## Supplementary Material

Supplementary Table 1 Primer sequences for encoding mRNA corresponding to 12 selected proteins quantified using real-time RT-PCR.

Supplementary Table 2 Abbreviations of the specific protein names in the protein–protein interaction network.
