# Supplementary material for: Proteomic Analysis of Fusarium oxysporum-Induced Mechanism in Grafted Watermelon Seedlings
Source: Front Plant Sci. 2021 Mar 4;12:632758. doi: 10.3389/fpls.2021.632758 (PMC7969889; doi:10.3389/fpls.2021.632758)
Supplement: Supplementary Table 1 — Primer sequences for encoding mRNA corresponding to 12 selected proteins quantified using real-time RT-PCR. [file Table_1.DOCX]

**TABLE S1 Primer sequences for the encoding mRNA corresponding to twelve selected proteins quantified using real-time RT-PCR**

| **Spot** | **Gene** | **Watermelon accession** | **Forward primer (5'-3' )** | **Reverse primer (5'-3' )** | **Product size (bp)** |
| --- | --- | --- | --- | --- | --- |
| L2 | universal stress protein | Cla97C05G088120 | TTAAGCCCGATGCTGAAACT | GCTTGCCAAGACCTCTGTTC | 168 |
| L6 | thioredoxin h | Cla97C08G158200 | AATTGCCGAGAAGTTCGATG | CAGCTCCACTTTGTCAGCAC | 100 |
| L9 | cytosolic glutamine synthetase | Cla97C05G093490 | TGTTAAGTGGCCCATTGGAT | GTACCGAGCAACCCACAGTT | 244 |
| L15 | thaumatin-like protein | Cla97C01G003090 | CTCGTATTTGGGCACGAACT | GTACCGTAGGCTTGGCACTG | 103 |
| L21 | hsp70-Hsp90 organizing protein 3-like | Cla97C10G187570 | AATCTGGACTGGCTGATGCT | GATCAGCGGTCAGTTTAGCC | 120 |
| L22 | Jasmonate-induced protein | Cla97C11G207000 | CTCTTCTGCTGCGTGTGTGT | TGCCGTATTGTCTGAAGCTG | 109 |
| L23 | L-ascorbate peroxidase T | Cla97C08G148570 | CTATTGGAGCCCATCAAGCA | GGTTCGGGTTTGTCCTCTCT | 137 |
| L28 | arginase | Cla97C06G109750 | TTGGTGATGTCCCTGTTCAA | TCCTCTTCCATCACCAGCTT | 100 |
| L31 | Ferredoxin--NADP reductase | Cla97C10G185280 | CCCTTGGTGACTTTGGTGAC | TCTTCACCTCTGCTCCAGGT | 138 |
| L35 | isoflavone reductase-like protein/ | Cla97C02G034000 | GTTGGTGCGGGTCAGTTAGC | ACGGCATGTCCACGATCAAC | 119 |
| *18SrRNA* | GenBank accession no. AB490410 | | AGCTTGAGAAACGGCTACCA | CGAAGAGCCCGGTATTGTTA | 105 |
