# Supplementary material for: Proteomic Analysis of Fusarium oxysporum-Induced Mechanism in Grafted Watermelon Seedlings
Source: Front Plant Sci. 2021 Mar 4;12:632758. doi: 10.3389/fpls.2021.632758 (PMC7969889; doi:10.3389/fpls.2021.632758)
Supplement: Supplementary Table 2 — Abbreviations of the specific protein names in the protein–protein interaction network. [file Table_2.DOCX]

**TABLE S2 Abbreviations of the specific protein names in the protein-protein interaction network**

| **Spot** | **Abbreviation** | **Protein** |
| --- | --- | --- |
| ***C metabolism*** | | |
| L14 | AT2G45290 | Transketolase-2 |
| L33 | MDH | Malate dehydrogenase, chloroplastic |
| ***N metabolism*** | | |
| L8/L9 | GLN1-1 | Glutamine synthetase cytosolic isozyme 1-1 |
| ***ROS metabolism*** | | |
| L23 | TAPX | Ascorbate peroxidase tAPX |
| L37 | APX1 | Ascorbate peroxidase APX1 |
| ***Energy metabolism*** | | |
| L13 | ATPD | ATP synthase subunit delta, chloroplastic |
| L24 | LOS2 | Enolase 2 |
| L27 | AT2G07698 | ATPase, F1 complex, alpha subunit protein |
| L30 | ATPC1 | ATP synthase gamma chain 1 |
| ***Protein biosynthetic*** | | |
| L1 | CYN | cyanate hydratase |
| L10 | OASB | Cysteine synthase |
| L11 | MOD1 | Enoyl-[acyl-carrier-protein] reductase [NADH] |
| L28 | ARGAH1 | Arginase 1 |
| L34 | MST1 | Thiosulfate/3-mercaptopyruvate sulfurtransferase 1 |
| ***Defense and stress*** | | |
| L6 | TRX5 | Thioredoxin H-type 5 |
| L35 | AT4G39230 | isoflavone reductase |
| ***Translation*** | | |
| L3 | ELF5A-1 | Eukaryotic translation initiation factor 5A (EIF-5A) |
| L12 | AT2G40010 | Ribosomal protein |
| L29 | RABE1b | Elongation factor Tu, chloroplastic |
| ***Photosystem*** | | |
| L31 | FNR1 | Ferredoxin--NADP reductase |
| L36 | AT2G44920 | Tetratricopeptide repeat (TPR)-like superfamily protein |
| L38 | PSBP-1 | Oxygen-evolving enhancer protein 2-1, chloroplastic |
| ***Signal transduction*** | | |
| L17 | GER3 | Germin-like protein |
| L18 | NDPK1 | Nucleoside diphosphate kinase family protein |
| ***Transport*** | | |
| L19 | PETC | Cytochrome b6-f complex iron-sulfur subunit |
